# Chicago Dental Society—Dr. W. W. Allport

**Published:** 1893-08

**Authors:** 


### Obituary.

### CHICAGO DENTAL SOCIETY—DR. W. W. ALLPORT.

The following Resolutions on the death of Dr. W. W. Allport were adopted by the Chicago Dental Society.

Whereas, In the death of Dr. W. W. Allport a leader in our profession has fallen, as a mark of our appreciation of his services and skill be it

*Resolved*, That in his death the dental profession has lost a member whose extraordinary skill as an operator placed him among the foremost dentists of the world. His work in promoting the highest interests of the profession will ever be conspicuous, and the prosperity enjoyed by younger members is due in a great measure to his achievements.

*Resolved*, That a copy of this preamble and resolutions be sent in proper form to the family of the deceased, and also to the International Dental Journal for publication.

Truman W. Brophy,
A. W. Harlan,
J. N. Crouse,
*Committee.*

